# A systematic review of observational studies on oxidative/nitrosative stress involvement in dengue pathogenesis

**Published:** 2015-09-30

**Authors:** Raimundo Castro, Hernando Samuel Pinzón, Nelson Alvis-Guzman

**Affiliations:** 1 University of San Buenaventura, Cartagena, Colombia.; 2 Children's Hospital Foundation Napoleon Franco Pareja, Cartagena, Colombia; 3 University of Cartagena, Cartagena, Colombia

**Keywords:** Dengue, severe dengue, dengue virus, oxidative stress, nitrosative stress, biological markers, systematic review

## Abstract

**Objective:**

Our objective was to systematically review the published observational research related to the role of oxidative-nitrosative stress in pathogenesis of dengue.

**Methods:**

We searched electronic databases (PubMed, EMBASE, The COCHRANE library, ScienceDirect, Scopus, SciELO, LILACS via Virtual Health Library, Google Scholar) using the term: dengue, dengue virus, severe dengue, oxidative stress, nitrosative stress, antioxidants, oxidants, free radicals, oxidized lipid products, lipid peroxides, nitric oxide, and nitric oxide synthase.

Articles were selected for review by title and abstract excluding letter, review, *in vivo* and *in vitro* studies, and duplicates studies. Selected articles were reviewed for study design, original purposes, sample size, main outcomes, methods, and oxidative-nitrosative stress markers values.

**Results:**

In total, 4,331 non-duplicates articles were identified from electronic databases searches, of which 16 were eligible for full text searching. Data from the observational studies originate from Asian countries (50%; 8/16), South American countries (31.2%; 5/16), and Central America and the Caribbean countries (18.8%; 3/16). Case-control study was the type of design most common in researches reviewed. The 1997 World Health Organization (WHO) dengue case classification criteria were used in all studies included in this review.

**Conclusions:**

Based on published data found in peer-reviewed literature, oxidative and nitrosative stress are demonstrated by changes in plasma levels of nitric oxide, antioxidants, lipid peroxidation and protein oxidation markers in patients with dengue infection. Additionally, elevated serum protein carbonyls and malondialdehyde levels appear to be associated with dengue disease severity.

## Abstract (es)

**Objetivo:**

Sistematizar las evidencias observacionales sobre la relación entre el estrés oxidativo-nitrosativo y la patogénesis del dengue.

**Métodos:**

Revisión sistemática de estudios observacionales en las bases de datos (PubMed, EMBASE, The COCHRANE library, ScienceDirect, Scopus, SciELO, LILACS via Virtual Health Library, Google Scholar) utilizando las siguientes palabras clave: dengue, dengue virus, severe dengue, oxidative stress, nitrosative stress, antioxidants, oxidants, free radicals, oxidized lipid products, lipid peroxides, nitric oxide y nitric oxide synthase. La selección inicial fue realizada a partir del título y resumen excluyéndose: cartas para editor, revisiones, estudios *in vivo*/*in vitro* y duplicados. A cada artículo seleccionado, se le revisó el diseño del estudio, objetivo, tamaño de la muestra, resultados principales y niveles plasmáticos de los marcadores de estrés oxidativo-nitrosativo.

**Resultados:**

De 4,331 publicaciones encontradas, 16 estudios cumplieron con los criterios de inclusión. El 50 % (8/16) de los estudios revisados fueron realizados en países de Sur América, Centro América y del Caribe. El diseño de casos y controles fue el más frecuente. El anterior sistema de clasificación de casos (OMS-1997) fue utilizado en todos los estudios incluidos en esta revisión.

**Conclusiones:**

El estrés oxidativo-nitrosativo se encuentra presente en el curso de la infección por virus dengue, demostrado por los cambios en las concentraciones plasmáticas de óxido nítrico, antioxidantes y marcadores de lipoperoxidación y de oxidación de proteínas. Por último, parece existir una asociación entre la elevación de los niveles plasmáticos de los carbonilos proteicos y malondialdehído con la severidad del dengue.

## Introduction

Dengue is a systemic viral infection with a significant socioeconomic and disease burden in many tropical and subtropical regions all over the world. Using cartographic approaches, one recent estimate indicates 390 million dengue infections per year (95% credible interval 284-528 million), of which 96 million (95% credible interval 67-136 million) manifest clinically (with any severity of disease) 1. 

The etiologic agent of this tropical disease is dengue virus (DENV), member of the family Flaviviridae, with four different antigenic serotypes (DENV-1 to -4). The DENV genome of plus strand RNA encodes three structural proteins (capsid, prM and envelope) and seven non-structural proteins (NS1, NS2a, NS2b, NS3, NS4a, NS4b y NS5) 2,3

According to the PAHO, in 2013, for America reported 2,386,836 cases of dengue; of which 37,898 (1.6%) corresponded to severe dengue, with case fatality rate 0.06. Colombia, Andean country, reported 127,219 cases of dengue (55% of cases reported in Andean Sub-region); of which 3,377 (81.1% of cases reported in Andean Sub-region) corresponded to severe dengue 4. 

Clinical and epidemiological observations showed that dengue disease severity may vary according to age, ethnicity, genetic factors, immune status and underlying disease. It may also depend on the co-circulation of DENV serotypes and reinfection by different DENV serotypes 5-9. In this respect, it has been proposed the involvement of DENV infection-derived oxidative stress on the severity of dengue. This is based on their ability to trigger the release of proinflammatory cytokines, including TNF-alpha, participating in collective action in the immunopathogenesis of dengue disease 10.

By definition, oxidative stress is an imbalance between pro-oxidants and antioxidants in favour of the pro-oxidants 11, 12. Instead, nitrosative stress is defined as an indiscriminate nitrosilation of biological molecules 13. In the absence of an appropriate compensatory response from endogenous antioxidant defense system, the activation of several stress-sensitive intracellular signaling pathways have been reported. This activation involves the production of gene products that can lead to cell death and/or pathophysiological conditions 13 -16.

The effects of DENV-derived oxidative stress and redox imbalance on human and animal cell cultures have been explored. The NO, ROS, and reactive nitrogen species-RNS levels, GSSG/GSH ratio, iNOS gene expression and phosphorylation of STAT-1 were increased during *in vitro* infection 17-26.

Recently, Olagnier *et al*., 27 have reported that nuclear factor-erythroid 2-related factor 2/Nrf-2 mediated oxidative stress response, iNOS signaling and production of NOS and ROS pathways were stimulated by DENV-2 infection of human monocyte-derived dendritic cells/Mo-DC. Also, a statistically significance decrease in SOD-2 mRNA levels was observed during treatment with ROS scavenger diphenyleneiodonium-DPI. In addition, these authors reported that DENV-2 infection was associated with NADPH oxidase-generated ROS accumulation.

These lines of evidence suggest that oxidative/nitrosative stress can be related to production of pathogenesis-related protein, increased susceptibility of mice to DENV infection, hemorrhage development in mice, proinflammatory cytokines and transcriptional factor expression, and DENV replication in various cell cultures.

Considering the aforementioned scenario, we performed a systematically review of observational studies evaluating the role of oxidative-nitrosative stress in pathogenesis of dengue. This review is important because understanding the involvement of oxidative and nitrosative stress in dengue pathogenesis could have potential implications for prognosis and treatment.

## Materials and Methods

### Search strategy

This review was guided by the standard PRISMA protocol (Preferred Reporting Items for Systematic Reviews and Meta-analysis) 28 and was registered on PROSPERO, an international database of prospectively registered systematic reviews in health and social care managed by Center for Review and Dissemination, University of York, on 12 November 2014; http://www.crd.york.ac.uk/PROSPERO (CRD42014014878). PubMed, EMBASE, The COCHRANE library, ScienceDirect, Scopus, SciELO, LILACS via Virtual Health Library, Google Scholar databases were searched for articles using a combination of descriptors to select the studies of interest.

### Study selection 

After finding previously published studies in the databases with the descriptors "dengue" OR "dengue virus" combined with "oxidative stress" OR "nitrosative stress" OR "antioxidants" OR "oxidants" OR "free radicals" OR "oxidized lipid products" OR "lipid peroxides" OR "superoxide dismutase" OR "thioredoxin reductase" OR "nitric oxide" OR "nitric oxide synthase", we performed an analysis on the inclusion/exclusion criteria. This electronic search strategy was supplemented by scanning the reference lists of all articles to identify additional studies that may have been missed during the initial search. 

As inclusion criteria, we used observational studies that evaluated oxidative-nitrosative stress in dengue pathogenesis. Exclusion criteria were impossible extraction of data, no control group, dates from mosquito cells cultures, the editorial, comments, case reports, letter to the editor, conference abstract, review articles, proteomics, *in vitro* and *in vivo* studies. When multiple publications from the same study population were available, we included the most recent publication. 

Additionally, two authors reviewed the studies independently in case of disagreement a third author was consulted. In this systematic review, there is no restriction regarding to language, publication period, patient age (children or adult), or study design.

### Data extraction strategy

Articles were selected for review by title and abstract. After reading full-text articles, we extracted data relating to study design, original purposes, sample size, main outcomes, methods, and oxidative stress markers values.

The systematic computerized literature search of published observational studies was carried out in June 2014.

## Results

### Identification of studies

**Figure 1.**
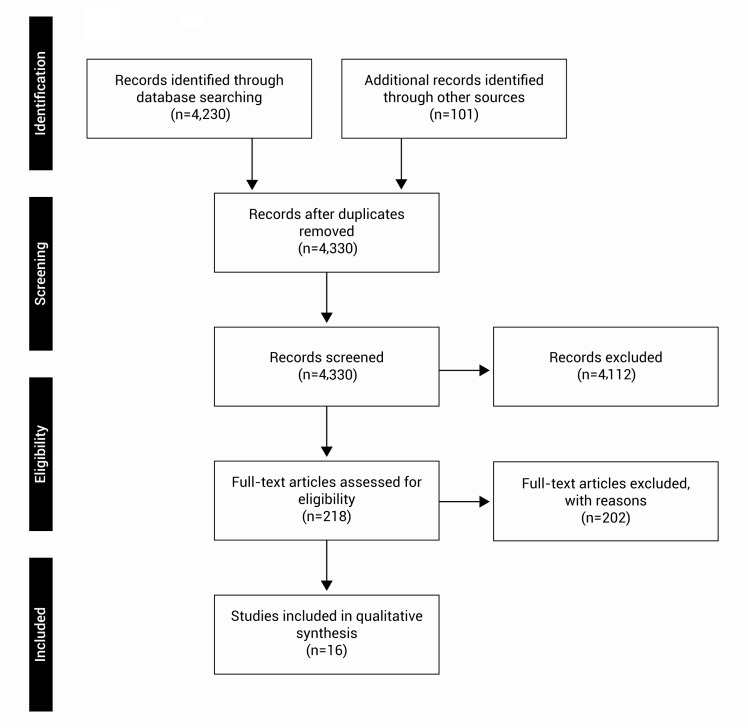
Flow diagram of studies included in the systematic review.

In total, 4,331 non-duplicates articles were identified from electronic databases searches, of which 16 were eligible for full text searching (Fig. 1). Table 1 presents the citation, definitions and characteristics of each included studies, respectively.

**Table 1.** Observational evidences of oxidative and nitrosative stress involvement in dengue pathogenesis

Study: author, Country, Reference	Study Design	Original purposes	Sample size	Outcomes
Ray, India, 50	Case-control	To evaluate the status of antioxidants, enzymes of hepatic and muscular origin and other biochemical indicators in children with dengue illness at admission	66 children with dengue	SOD: ↑Patients vs. Controls (*p* <0.005)
			(45 days to 12 yrs )	
			25 healthy children	GPx: ↓ Patients vs. Controls (*p* <0.005)
			Follow-up period: none	
Valero, Venezuela, 34	Case-control	To determine the serum concentration of NO in patients with DF and DHF	105 patients with dengue	Nitric oxide:
			53 healthy individuals	↑ DF vs. DHF (p <0.01)
			(Age-matched controls)	↑ DF vs. Controls (p <0.01)
			Follow-up period: none	
Gil, Cuba, 39	Case-control	To study the status of some oxidative stress markers in serologically confirmed adults dengue patients comparing with those observed in healthy individuals	22 adults with dengue	TAS (day 7): ↑ Patients vs. Controls (*p* <0.05)
			(21 to 58 yrs)	
			22 healthy individuals	PP - SOD - MDA/4-HAD (days 3-5-7): ↑ Patients vs. Controls (*p* <0.05)
			(Sex- and age-matched controls)	
			Follow-up period: 7 days	THs - GPx (days 3-5-7): ↓ Patients vs. Controls (*p* <0.05)
Klassen, Guatemala, 40	Case-control	To report data of several micronutrients with antioxidants in patients with classic dengue fever during the acute phase and convalescence from the disease and from appropriate reference control subjects	9 adults with dengue	Retinol: ↓ Patients (DI+DII) vs. Controls (*p* <0.05)
			(18 to 68 yrs)	Retinol (patients): ↓ DI vs. DIII (*p* <0.05)
			12 healthy individuals	b-carotene: ↓ DII vs. DIII (p= 0.03)
			Follow-up period:	GSH: ↓ DII vs. DI (*p*= 0.02)
			DI: admission to the hospital	GSH (at DIII): ↓ Patients vs. Controls (*p* <0.05)
			DII: 5 days after admission (discharge)	TBARS (DI+DII): ↑ Patients vs. Controls (*p* <0.05)
			DIII: 7 days after admission	TBARS (at DIII): ↑ Patients vs. Controls (*p* <0.05)
				TAS (DI vs. DII): ↓ Patients vs. Controls (p= 0.01)
Trairatvorakul, Thailand, 35	Case-control	To correlate nitric oxide levels in three groups with different severity ranging from DF, DHF I/II, to DSS, and to compare the results with a control group	110 children with dengue	Nitric oxide:
			(<15 yrs)	↓ Patients vs. Controls (p <0.05)
			38 healthy children	↑DF vs. DHF I/II (p <0.05)
			(Age-matched controls)	↓ DHF I/II vs. DSS (p <0.05)
			Follow-up period: none	
Rojas, Colombia, 51	Cross-sectional study	To evaluate the association between the levels of glutathione peroxidase and the manifestations and complications of dengue	161 patients with dengue	GPx:
			(6 to 85 yrs)	↑ Patients with hemorrhages vs Patients without hemorrhages (p= 0.03)
Chareonsirisuthigul, Thailand, 25	Cross-sectional study	To investigate the effect of antibody-dependent enhancement infection on pro- and anti-inflammatory cytokines production	60 children with dengue	Nitric oxide (secondary DHF):
			(5 to 10 yrs)	↓ Fever day vs. Convalescent day (p <0.05)
Soundravally, India, 48	Case-control	To assess whether oxidative stress induced changes in plasma protein can be an early predictor of severe dengue disease	80 patients with dengue (26-53 yrs)	PCO:
				↑ Patients vs. Controls (p <0.001)
			63 healthy individuals	↓ DF vs. DHF (p <0.05)
				↓ DF vs. DSS (p <0.05)
				↓ DHF vs. DSS (p <0.05)
			(Sex- and age-matched controls)	PBSH:
				↓ Patients vs. Controls (p <0.001)
			Follow-up period: none	↑ DF vs. DHF (p <0.05)
				↑ DF vs. DSS (p <0.05)
Soundravally, India, 41	Case-control	To investigate the association of lipid peroxidation and protein carbonylation with thrombocytopenia in the different clinical spectrum of dengue infection	80 patients with dengue (26-53 yrs)	MDA (3-5-7 days):
				↑ Patients vs. Controls (p <0.05)
			63 healthy individuals	↓ DF vs. DSS (p <0.05)
				MDA (5-7 days):
			(Sex- and age-matched controls)	↓ DF vs. DHF (p <0.05)
				PCOs (7 day):
			Follow-up period:	↑ Patients vs. Controls (p <0.001)
				TAS (3-5-7 days):
			7 days	↓ DSS vs. Controls (p <0.05)
Mendes-Ribeiro, Brazil, 36	Case-control	To investigate the effects of DF on the platelet L-arginine-NO pathway, platelet function and an inflammatory acute-phase protein fibrinogen	16 patients with DF	Total rates of _L_-arginine transport and _L_-arginine transport via system y^+^L:
			(35 ± 4 yrs)	↑ DF vs. Controls (p <0.05)
			18 healthy controls	Total NOS activity:
			(Age-matched controls)	↑ DF vs. Controls (p <0.05)
			Follow-up period: none	
Seet, Singapore, 45	Case-control	To investigate oxidative stress during dengue infection	28 patients with dengue	Plasma HETEs:
			(23-68 yrs)	↑ Febrile stage vs. Controls (p <0.05)
				↑ Febrile stage vs. Convalescent stage (p <0.05)
			28 healthy controls	COPs:
				↓ Febrile stage vs. Controls (p <0.05)
			(Age-matched controls)	Urinary F_2_-IsoPs:
				↑ Febrile stage vs. Controls (p <0.05)
			Follow-up period: 25 days	↑ Febrile stage vs. Convalescent stage (p <0.05)
Lee, Singapore, 46	Case-control	To provide further insight into the relation between lipid oxidation products and human diseases	35 patients with dengue (25-86 yrs)	Plasma free F_2_-IsoPs:
				↑ DF onset vs. Controls (p <0.01)
				↑ DF onset vs. DF recovery (p <0.01)
			47 healthy controls	Urinary F_2_-IsoPs:
				↑ DF onset vs. Controls (p <0.01)
				↑ DF onset vs. DF recovery (p <0.01)
			(Age-matched controls)	Plasma total HETEs:
				↑ DF onset vs. Controls (p <0.01)
				↑ DF onset vs. DF recovery (p <0.01)
			Follow-up period: none	COPs:
				↓ DF onset vs. Controls (p <0.05)
				↓ DF onset vs. DF recovery (p <0.05)
Levy, Venezuela, 28	Cross-sectional study	To compare the serum levels of IL-6, TNFa, IL-1b, NO, CRP, C3 and apoptosis in DENV-infected patients and in monocytes/macrophages cultures	36 patients with DF	Nitric oxide:
				↑ DF vs. Controls (p <0.001)
			(3-53 yrs)	↑ DF vs. DHF (p <0.001)
				↑ Primary infection vs. Controls (p <0.001)
			34 patients with DHF	↑ Secondary infection vs. Controls (p <0.001)
			(3-53 yrs)	↓ Primary infection vs. Secondary infection (p <0.001)
Matsuura, Brazil, 37	Case-control	To investigate _L_-arginine transport as well as activity and expression of iNOS in DHF platelets	23 patients with DHF (33±14 yrs)	_L_-arginine transport via system y^+^L:
			25 healthy individuals	↑ DHF vs. Controls (p <0.05)
			(Age-matched controls)	Total NOS activity:
			Follow-up period: none	↑ DHF vs. Controls (p <0.05)
Gil, Cuba, 44	Case-control	To study the status of an extensive array of redox indexes as proposal of an integral and dynamic characterization of redox status	22 patients with dengue	MDA - HPO - SOD - PP: ↑ Patients vs. Controls (*p* <0.05)
			(18-84 yrs)	
			194 healthy individuals	GSH:
			(Age-matched controls)	↓ Patients vs. Controls (p <0.05)
			Follow-up period: 7 days	
Soundravally, India, 13	Case-control	To evaluate the levels of plasma MDA, TNF-a and IFN-g during defervescence, in all the three clinical groups of dengue compared to uninfected blood samples	81 patients with dengue	MDA:
			(16-67 yrs)	↓ DF vs. DHF (p <0.001)
			30 healthy individuals	↓ DF vs. DSS (p <0.001)
			(Sex- and age-matched controls)	↓ DHF vs. DSS (p= 0.017)
			Follow-up period: none	

DF: dengue fever; DHF: dengue hemorrhagic fever; SOD: superoxide dismutase; GSH: glutathione; GPX: glutathione peroxidase; ALB: albumin; CAT: catalase; MDA: malondialdehyde; PCO: protein carbonyl; HETEs: hydroxyl-eicosatetraenoic acid; COPs: cholesterol oxidative products; F2-ISOPs: F2-isoprostanes; TAS: total antioxidants status; HPO: hydroperoxides; PP: peroxidation potential; THs: total hydroperoxides.

### Overview of included studies

**Country of origin.** Data from the observational research originate from India (n= 4), Thailand (n= 2), Singapore (n= 2), Cuba (n= 2), Guatemala (n= 1), Brazil (n= 2), Venezuela (n= 2), and, Colombia (n= 1). 

**Study design**. Case-control study was the type of design most common in studies reviewed.

**Age.** The observational studies could be classified by the age range of participants: children (under 10 yrs, n= 1), adolescents (10 to 19 yrs, n= 1), children and adolescents (n= 1), adults (greater 19 yrs, n= 7), adolescents and adults (n= 3), children, adolescents and adults (n= 2), and age range: not reported (n= 1).

**Follow-up time.** Approximately 63% of observational studies reported a 7-day follow-up period. About a quarter of the included studies did not report follow-up of participants.

**Dengue case classification system**. The 1997 World Health Organization (WHO) dengue case classification criteria were used in all studies included in this review. 

As indicated in Table 2, lipid peroxidation products (malondialdehyde-MDA, and hydroperoxides-THs), protein carbonyls (PCOs), antioxidant enzymes (superoxide dismutase-SOD, and glutathione peroxidase-GPx), nitric oxide, and reduced glutathione (GSH) levels were determined spectrophotometrically or spectroflurometrically using commercial and non-commercial assays. The most analyzed oxidative-nitrosative stress markers were lipid peroxidation products (35.7%; 10/28), antioxidant enzymes (17.9%; 5/28), and nitric oxide (10.7%; 3/28). 

**Table 2.** Comparison of methods used and values reported in reviewed studies.

Markers	Methods	Reported values	References
NO	Spectrophotometric detection		
	Absorbance at 540 nm. (Griess reagent)	DF cases (DENV-1): 57.4 ± 3.02 mM	34
		DF cases (DENV-4): 55.6 ± 2.25 mM	
		DHF cases (DENV-2): 13.87 ± 2.05 mM	
		DHF cases (DENV 4): 14.33 ± 1.25 mM	
		Controls: approx. 25 mM	
	Absorbance at 540 nm. (Commercial kit, Griess method)	DF cases: approx. 40 mM	28
		DHF cases: approx. 22 mM	
		Controls: approx. &lt;10 mM	
		DF cases: 124.94 ± 36.79 mM	35
		DHF I/II cases: 99.69 ± 33.42 mM	
		DHF III/IV cases: 120.63 ± 46.26 mM	
		Controls: 168.18 ± 24.10 mM	
MDA	Absorbance at 532 nm. (Satoh method)	Dengue cases: 4.9 ± 0.9 mM	13
		Controls: 1.9 ± 0.4 mM	
	Absorbance at 586 nm. MDA + 4-HDA. (Commercial kit)	Dengue cases: approx. 10-18 mM	39
		Controls: approx. &lt;10 mM	
	Difference in absorbances at 532 and 572 nm. (Jentzsch method)	Dengue cases: 0.624 ± 0.144 mM	40
		Controls: 0.450 ± 0.116 mM	
	Absorbance at 532 nm. (Satoh method)	Dengue cases: approx. 2-8 mM	41
		Controls: approx. 2 mM	
THS	Absorbance at 560 nm. Oxidation of Fe(II) to Fe (III) by THs. (Commercial kit)	Dengue cases: approx. 50-150 mM	39
		Controls: approx. 200-280 mM	
	Absorbance at 560 nm. Oxidation of Fe(II) to Fe (III) by THs	Dengue cases: 119.6 ± 34.61 mM	44
		Controls: 70.3 ± 13.9 mM	
PCOs	Absorbance at 366 nm. (Method of Levine)	Dengue cases: approx. 3.0-8.0 nmol/mg protein	41
		Controls: approx. 2.0-2.5 nmol/mg protein	
		DF cases: 4.98 ± 0.47 nmol/mg protein	48
		DHF cases: 5.95 ± 0.61 nmol/mg protein	
		DSS cases: 6.66 ± 0.70 nmol/mg protein	
		Controls: 1.97 ± 0.56 nmol/mg protein	
SOD	Absorbance at 505 nm. (commercial kit)	DENV cases: approx. 1.8-3.0 U/ml	39
		Controls: approx. 1.2-1.7 U/ml	
		DENV cases: 2.26 ± 0.71 U/mg Hb	44
		Controls: 1.41 ± 0.72 U/mg Hb	
	Absorbance at 480 nm. (Epinephrine method)	DF: 26.85 ± 8.13 U/ml	50
		DHF: 23.17 ± 9.98 U/ml	
		DSS: 24.86 ± 9.22 U/ml	
		Controls: 2.31 ± 0.97 U/ml	

NO: Nitric oxide; MDA: Malondialdehyde; 4-HAD: 4-hydroxyalquenals; TBARS: Thiobarbituric acid reactive substances; THs: Total hydroperoxides; PCOs: protein carbonyls; SOD: Superoxide dismutase; DF: dengue fever; DHF: dengue hemorrhagic fever; DSS: dengue syndrome shock; UV: ultraviolet.

## Discussion

To our knowledge, no previous reviews on the involvement of oxidative-nitrosative stress in dengue pathogenesis have been performed. In the present systematic review, 16 articles concerning this subject were included.

The role of nitric oxide as strong immunomodulator and its association with viral infections, both *in vivo* and *in* *vitro*, have been clearly demonstrated 29, 30 but there are incongruous results in some studies in DENV-infected patients. For example, although elevated serum levels of nitric oxide have been observed in sera of patients with dengue fever (DF) in comparison to age-matched healthy controls 25, 31 or to patients with dengue hemorrhagic fever (DHF) 25, Trairatvorakul *et al*. 32, reported that serum nitric oxide levels were significantly higher in healthy children than in DF or DHF patients. These studies were characterized by the low case/control ratio ((1:0.5).

Now, the two first reports are consistent with high total nitric oxide synthase activity and high L-arginine transport via system y^+^L reported in platelet obtained from DF and DHF patients compared with healthy adult controls 33,34.

A study by Thai investigators reported that nitric oxide levels of primary and secondary DF, and primary DHF were not significantly different on the fever, defervescent or convalescent days in plasma of 37 DENV-infected children, while the level of serum nitric oxide was significantly lower on the fever day than on the defervescent day among children with secondary DHF. In addition, serum nitric oxide levels had an inverse correlation with the level of DENV viraemia in patients with secondary DHF 22, but have not been linked to viral serotype infection 31.

These observations are consistent with the findings of Yen *et al*., 17, who reported a temporal coincidence between iNOS upregulation and free radical production with hemorrhage development in DENV-infected mice.

Blood nitrite and nitrate levels are frequently assessed as an index of systemic nitric oxide production. The spectrophotometric assay based on the Griess reagent is the most commonly used method to determine nitrite/nitrate concentration in biological matrixes 35. In the Table 2, the contrasting results may be explained by differences in nitrate reduction and deproteinization methods used or by differences in characteristics of populations studied.

As regard lipid peroxidation products as biomarkers for oxidative stress, several studies have found high serum levels of MDA and 4-hydroxyalkenals (4-HAE) in dengue patients in comparison to healthy adult controls 10, 36 -38. Furthermore, others reported high serum MDA concentrations in DHF and dengue shock syndrome (DSS) patients than in DF patients. They also reported a positive correlation between serum MDA and TNF-α levels in all DENV-infected patients 10. 

Interestingly, a recent study reported that DENV-2-infected mice showed alterations in oxidative stress by increasing the level of malondialdehyde (MDA) 18. These data together provide direct evidence for use end products of lipid peroxidation as prognostic biomarker in dengue disease.

Similar concentrations of MDA were reported using Satoh method 10, 38. Both patients and healthy controls, Klassen *et al*. 37, reported lower serum levels of MDA using Jentzch correction method. Instead, Gil *et al*. 36, reported high serum MDA concentrations in dengue patients. This sensible difference could be explained by detection of MDA in combination with 4-HAE and/or sensitivity and reproducibility of the method 39. 

Although THs levels are considered a measure of overall oxidative damage 40, there are no consistent pattern of THs in dengue disease. Gil *et al*., 36 reported that serum THs concentrations were significantly decreased in dengue patients than in healthy adults. By contrast, the same group researchers recently reported elevated serum THs levels in dengue patients in comparison to adult controls 41. In dengue cases, unlike controls, both studies reported similar serum THs concentrations.

Other oxidized lipid biomarkers have been studied. For example, isoprostanes (IsoPs), hydroxyeicosatetraenoic acid products (HETEs) and cholesterol oxidation products (COPs). 

During febrile stage of dengue infection, high concentrations of urinary F_2_-IsoP and plasma HETEs levels have been detected in comparison with convalescent stage and also with healthy adults 42. No significant differences were found in: (i) total F_2_-IsoPs levels in all three dengue infection phases in comparison to adult controls 42, 43, (ii) urinary F_2_-IsoPs and plasma HETEs levels between defervescence and convalescence phases 42, and (iii) total F_2_-IsoPs concentrations between onset of DF and recovery stage of DF. However, elevated plasma total HETEs levels and reduced COPs levels were observed in onset of DF than in recovery stage of DF or in controls 43.

With respect to PCOs content, it has been used as an important biomarker of protein oxidative damage in oxidative stress-related diseases in humans 44. Elevated serum PCOs levels have been reported in dengue patients in comparison to adult controls. Moreover, elevated serum PCOs concentrations were associated with dengue disease severity 38, 45. In both studies reported similar serum PCOs levels using method of Levine.

By definition, oxidative stress also involves the antioxidant defensive system which is formed by ROS protective enzymes (SOD, GPx, glutathione reductase-GR, Catalase-CAT, thioredoxin-TRX system, antioxidant nutrients, etc.), reactive oxygen species (ROS) scavengers (GSH, albumin, ascorbic acid, and uric acid), and sequestration of transition metal ions (transferrin, ferritin, metallothionein and ceruloplasmin) 46.

At all ages, elevated serum SOD levels and reduced GPx levels have been reported in dengue patients in comparison with controls 36, 41, 47. By contrast, no differences were found in plasma antioxidants nutrients (except of retinol) concentrations between dengue patients and adult controls, but in this study only 9 patients and 12 healthy controls were evaluated 37. 

Results of one study have shown that serum GPx levels were associated with spontaneous bleeding events and with serum triglycerides levels in dengue patients. This finding suggests that the intensity of oxidative stress can influence the clinical presentation of dengue 48. In this regard, a recent study reported that increased triglycerides levels were observed mainly in severe dengue patients 49. Based on these data, it is likely that serum lipid profile in DENV-infected patients plays an important role in dengue severity. 

Free radical production and alterations in antioxidants status occur during dengue disease 37. The major antioxidant defense system appears to function by scavenging radical free radical. Non-enzymatic scavenger like GSH has been shown to be an effective protector against oxidative damage 46. There are differences in the reports on the comparison of GSH concentrations in dengue patients and healthy controls. Gil *et al*. 36, and Klassen *et al*. 37, reported that serum GSH levels were not significantly different between patients and controls, whereas Gil *et al*. 41 reported that serum GSH concentrations were significantly lower in dengue patients than in healthy adults controls. It is important to note two aspects: (i) these three researches were conducted in Central America and the Caribbean countries and (ii) the number of patients studied was small (n (22).

Despite the limitations of systematic reviews of observational studies, given the evidences presented here, oxidative-nitrosative stress is demonstrated by changes in plasma levels of nitric oxide, antioxidants, lipid peroxidation and protein oxidation markers in patients with dengue infection. 

Additionally, elevated serum PCOs and MDA levels appear to be associated with dengue disease severity, expressed in terms of the 1997-WHO dengue case classification system. Accordingly, more data are needed to establish an association between oxidative-nitrosative stress and dengue severity in the context of 2009-WHO dengue classification scheme.

## Conclusions

Based on published data found in peer-reviewed literature, oxidative and nitrosative stress are demonstrated by changes in plasma levels of nitric oxide, antioxidants, lipid peroxidation and protein oxidation markers in patients with dengue infection. Additionally, elevated serum protein carbonyls and malondialdehyde levels appear to be associated with dengue disease severity.

There have been many studies that target the severity of dengue infections. However, our understanding is not complete yet. Better understanding of relation between oxidative-nitrosative stress and dengue pathogenesis will lead us to developing better prognostic strategies of this pathology.
